# COVID-19 in Romania: What Went Wrong?

**DOI:** 10.3389/fpubh.2021.813941

**Published:** 2021-12-17

**Authors:** Stefan Dascalu, Oana Geambasu, Catalin Valentin Raiu, Doina Azoicai, Emilian Damian Popovici, Cristian Apetrei

**Affiliations:** ^1^Department of Zoology, University of Oxford, Peter Medawar Building for Pathogen Research, Oxford, United Kingdom; ^2^Avian Influenza Research Group, The Pirbright Institute, Surrey, United Kingdom; ^3^T. H. Chan School of Public Health, Harvard University, Boston, MA, United States; ^4^Faculty of Business and Administration, Department of Public Administration, University of Bucharest, Bucharest, Romania; ^5^Department of Epidemiology, School of Medicine, Grigore T. Popa University of Medicine and Pharmacy, Iasi, Romania; ^6^Department of Epidemiology, School of Medicine, Victor Babeş University of Medicine and Pharmacy, Timişoara, Romania; ^7^Regional Centre of Public Health, Timişoara, Romania; ^8^Division of Infectious Diseases, School of Medicine, University of Pittsburgh, Pittsburgh, PA, United States; ^9^Department of Infectious Diseases and Immunology, Graduate School of Public Health, University of Pittsburgh, Pittsburgh, PA, United States

**Keywords:** public health, COVID-vaccine, COVID-19, SARS-CoV-2, Romania, public health policy, vaccination campaign

A catastrophic fourth wave of the COVID-19 pandemic in Romania raised international concern due to a rapid surge in the number of infections and the high associated mortality. A country of approximately 19 million inhabitants, Romania recorded close to 20,000 daily infections, with more than 500 daily deaths, by mid-October 2021 (1). Consequently, the WHO sent experts to Romania to evaluate the ongoing situation, including the status of the COVID-19 vaccination campaign, and to help with an action plan. Here, we provide explanations for this dramatic reality using information from previously published academic analyses, the authors' personal involvement in the Romanian COVID-19 mitigation efforts, and press articles which describe the evolution of the pandemic in Romania.

Similar to other EU countries, COVID-19 vaccines (both mRNA- and adenovirus-based) have been widely available to Romanians (2). Vaccination began on 27 December 2020, after emergency use authorization was granted by the European Medicines Agency (2). At the time, national surveys were indicating that only around 30% of Romanians would be willing to receive a vaccine against COVID-19. During the first few months, the vaccination campaign progressed as planned, but then it stalled. By the surge of the fourth wave, only ≈30% of Romanians were fully immunized, one of the lowest COVID-19 vaccination coverages in Europe, and the main reason for this epidemiological crisis (1). The factors driving this failure are multiple and intricate: (a) economic and social, with incomplete implementation of prevention measures and premature relaxation of restrictions, politically-driven and unsupported by the progress of the vaccination campaign; (b) insufficient support for the vaccination campaign, which was not linked to other preventive actions, lacked appropriate funding and resources, and only received minimal backing from top governmental authorities (3); (c) a hyper-politicization of COVID-19 public health measures, in the context of two rounds of general and local elections, plus internal elections in several major political parties, which resulted in a triumphalist rather than realistic assessment of the epidemic, inducing a false sense of security in the general public (4); (d) Chronic governmental instability, which, even prior to the pandemic, severely eroded people's trust in state authorities: over the last 6 years alone, Romania had eight prime ministers (average tenure: 273 days) and ten health ministers (average tenure: 218 days) (5); (e) in March 2020, the then newly-appointed government implemented severe restrictions during the lockdown and state of emergency (including mandatory hospitalization of asymptomatic cases), leading to a general uproar and constant transgressions of the prevention rules (6); (f) insufficient testing and tracking, resulting in disease underdiagnosis and incorrect assessments of the prevalence levels in the population, and of the main routes of viral spread (2); (g) throughout the pandemic, under the pretext of presenting “balanced viewpoints”, major news outlets generously featured representatives of the anti-vaccine movement and conspiracy theory advocates almost on a daily basis (7); (h) a high proportion of healthcare professionals refused vaccination (8); (i) the lack of administrative and judicial sanctions for perpetrators of misinformation, particularly those with academic credentials and medical degrees, contributed to a general mistrust of epidemic control measures that was deeply embedded in public perception; (j) relatedly, there was an absence of systematic consultations with major stakeholders in society, particularly academics and religious institutions and faith-based communities (9). As >80% of Romanians adhere to the Romanian Orthodox Church (ROC), harnessing the influence of the ROC could have greatly enhanced the outcomes of public health measures. However, throughout the pandemic, few, if any, consultations between state authorities and ROC representatives occurred, often leading to mixed responses (9). Indeed, very few influential bishops publicly endorsed vaccination.

As an overall result, public trust was catastrophically eroded, with the consequence that Romania entered the fourth epidemic wave with no restrictions, an insufficiently vaccinated population, and a completely divided society. In addition to these factors, some chronic deficiencies relating to both medical and socio-political factors contributed to this perfect storm. First, Romania has the lowest healthcare GDP expenditure per capita in the EU, and the healthcare system was severely underprepared for the pandemic (6). Second, political instability led to multiple inconsistencies in healthcare policies, including the absence of a legislative framework for vaccinations (10). This instability is also reflected in the education sector, which was also subject to multiple reforms over the last 30 years, with unavoidable consequences on public education. Furthermore, emigration of educated professionals has been massive and constant over the last 30 years (11). Altogether, these factors contribute to one of the lowest vaccination coverages for vaccine-preventable diseases in Europe, with recurrent outbreaks such as the severe measles epidemic of 2016 (6, 10).

By the time COVID-19 vaccines became available, all these issues had remained unaddressed, while realistic and balanced public debates on the benefits of a successful vaccination campaign were long overdue (2). As such, any attempts to reach the necessary vaccination coverage were severely hindered.

The catastrophic fourth wave of the COVID-19 pandemic in Romania tragically illustrates the impending need to address vaccine hesitancy in the general population, as well as preparing the healthcare system to successfully respond to a national emergency.

One of the masterpieces of the new wave of Romanian cinema is called *Too Late*. Indeed, in Romania, the fourth wave of the COVID-19 pandemic has generated a tragedy that the country acknowledged, sadly, *too late*.

## Author Contributions

SD, OG, CR, DA, EP, and CA: collected data and established the content of the manuscript. SD and CA: wrote the manuscript. OG, CR, DA, and EP: reviewed and corrected the manuscript. All authors contributed to the article and approved the submitted version.

## Funding

SD was supported by the BBSRC, grant number BB/M011224/1. CA was supported by grants R01AI119346, R01DK130481, R01DK113919, R01DK119936, and R01DK131476 from the National Institutes of Health (NIH)/National Institute of Allergy and Infectious Diseases (NIAID) and the National Institute of Diabetes and Digestive and Kidney Diseases (NIDDK). Funders had no role in study design, data collection and analysis, decision to publish, or preparation of the manuscript.

## Author Disclaimer

The content of this publication does not necessarily reflect the views or policies of the Department of Health and Human Services, nor does mention of trade names, commercial products, or organizations imply endorsement by the U.S. Government.

## Conflict of Interest

The authors declare that the research was conducted in the absence of any commercial or financial relationships that could be construed as a potential conflict of interest.

## Publisher's Note

All claims expressed in this article are solely those of the authors and do not necessarily represent those of their affiliated organizations, or those of the publisher, the editors and the reviewers. Any product that may be evaluated in this article, or claim that may be made by its manufacturer, is not guaranteed or endorsed by the publisher.
